# Machine learning-based guilt detection in text

**DOI:** 10.1038/s41598-023-38171-0

**Published:** 2023-07-15

**Authors:** Abdul Gafar Manuel Meque, Nisar Hussain, Grigori Sidorov, Alexander Gelbukh

**Affiliations:** 1 Instituto Politécnico Nacional (IPN), Centro de Investigación en Computación (CIC), Mexico City, Mexico; 2 Faculdade de Economia e Gestao, Catholic University of Mozambique, Beira, 2100 Mozambique

**Keywords:** Computer science, Computational science

## Abstract

We introduce a novel Natural Language Processing (NLP) task called guilt detection, which focuses on detecting guilt in text. We identify guilt as a complex and vital emotion that has not been previously studied in NLP, and we aim to provide a more fine-grained analysis of it. To address the lack of publicly available corpora for guilt detection, we created VIC, a dataset containing 4622 texts from three existing emotion detection datasets that we binarized into guilt and no-guilt classes. We experimented with traditional machine learning methods using bag-of-words and term frequency-inverse document frequency features, achieving a 72% f1 score with the highest-performing model. Our study provides a first step towards understanding guilt in text and opens the door for future research in this area.

## Introduction

In this study, we introduce guilt detection, a novel task in Natural Language Processing aimed at detecting guilt in text. We also developed a dataset and a set of baseline classifiers for this task. Guilt is a complex emotion that arises when individuals contemplate past wrongdoings or failings to uphold their own moral standards^1^. It is frequently felt when people feel responsible for wrongdoing or harm to others, whether real or imagined^2^. It is often accompanied by a desire to correct any perceived interpersonal flaws^3^.

In text processing, guilt can be detected through linguistic markers that indicate a sense of responsibility or remorse for past actions or events. These markers may include words or phrases that express regret, such as “sorry” or “I wish I had done things differently,” or those that indicate a sense of responsibility or culpability, such as “I should have known better” or “I feel responsible for what happened.” It is important to note that guilt can manifest more subtly in text, such as through indirect or ambiguous language, self-deprecation, or avoidance of specific topics or individuals^4,5^. As such, a comprehensive understanding of guilt in text requires a nuanced approach that considers both explicit and implicit linguistic markers.

Furthermore, the experience of guilt can be influenced by cultural and societal norms and individual differences in personality, cognitive processing, and emotional regulation^6^. Therefore, any analysis of guilt in texts must also consider these factors and their potential impact on the expression and interpretation of guilt-related language. Detecting and analyzing guilt in text presents a challenging but essential task for natural language processing research, with potential applications in mental health, social media analysis, and criminal justice.

Sharing emotional experiences is a crucial component of the emotional process (the psychological and physiological events that occur when an individual experiences an emotion. It involves the cognitive interpretation and subjective experience of an emotion, as well as the behavioral and physiological responses that result from it.^7^), as observed by^8^; furthermore, in^9^, the authors found that sharing emotional experiences on the internet has become a crucial part of daily life **on** contemporary cultures. This can explain the popularity of emotion-related research in many scientific disciplines, including computer science, particularly Natural Language Processing (NLP), to determine the presence of, assess the intensity and polarity of, and compare various emotional experiences shared in the form of written text. Despite the considerable interest in emotion detection, there is still a sizable research vacuum regarding the in-depth examination of particular emotions, such as guilt.

Guilt comes in many forms, namely anticipatory, existential, and reactive guilt^2^, and it is frequently felt when people feel responsible for wrongdoing or failing to uphold their own moral standards^1^; as a result, it frequently indicates an understanding of the experiences of others and a desire to correct any perceived interpersonal flaws^3^. As with other self-conscious emotions, when in excess, guilt can have negative implications for one’s mental health^10^, thus attracting the interest of researchers in various disciplines, from Psychology to neuroscience to Computer Science, in the latter, particularly as a sub-field in Natural Language Processing, guilt has been featured as a class in Emotion Detection sub-tasks in numerous NLP research works.

Guilt is a complex emotion that is crucial in our everyday lives. It has been studied extensively in psychology and philosophy but has not yet received the same attention in Natural Language Processing (NLP). In social media analysis, detecting guilt in user-generated content can help social media platforms develop more targeted and effective interventions for users experiencing negative emotions. In legal contexts, guilt detection can be used to evaluate the truthfulness of legal statements and identify potential suspects.

Considering the mental health implications of excessive guilt, as mentioned in^10^ and supported by numerous research studies that link guilt to suicidality in clinical populations^3,11,12^, there is clear importance and value in studying ways to identify if, when, and how an individual experiences guilt.

Despite the potential applications, to our knowledge, no extensive research in NLP focuses on guilt detection as a primary subject of study. Previous studies such as^13,14^ have only included guilt in a multi-class emotion detection task. Our paper aims to fill this research gap by building a binary guilt detection dataset and evaluating the performance of traditional and deep learning models on this dataset.

One could argue that the existing multiclass emotion detection datasets that include guilt as one of the classes already feature some form of guilt detection. However, those studies focused on detecting multiple emotions, not specifically on guilt. By creating a binary guilt detection dataset and developing models specifically for detecting guilt, this study provides a more focused approach to understanding and detecting this particular emotion. Additionally, the existing datasets may not have had sufficient examples of guilt instances or may have had noise and bias from the other emotions included in the dataset. Creating a dedicated guilt detection dataset helps to address these issues and provides a more accurate and reliable means of detecting guilt.

In summary, our paper’s novelty lies in focusing on guilt detection as a primary subject of study in NLP and developing a binary guilt detection dataset. We believe this research can contribute to better understanding of guilt as an emotion and its applications in various industries.

The following are the main contributions of this paper:

- A study of guilt detection from text using NLP techniques
- Development of a multi-source dataset for binary guilt detection,
- Development of baseline models for the proposed dataset,
- An in-depth analysis of the dataset and models

The remainder of the paper consists of the following sections: “Literature review,” which showcases works on emotion detection, particularly those that focus on guilt; “Dataset development,” which describes the techniques used to acquire and build the dataset; “Benchmark experiments,” which presents the setups for the baseline model experiments; and “Results and discussion,” where an initial analysis of the baseline results is provided. Finally, the paper concludes with “Conclusion and future work,” which suggests possible future directions for research on guilt.

## Literature review

To the best of our knowledge, there has not been any comprehensive research work done targeting guilt, nor is there any dataset dedicated to guilt emotion, at least not one that is publicly available. Although guilt itself has not been the main subject of research, it has been featured in various studies, such as in^13,15^, where the authors explore a way and propose an approach to detect different types of emotions, using a Common Sense Knowledge EmotiNet, which they extended and improved in^16^.

In this section, we present related work regarding both datasets and techniques for Emotion Detection in general and fine-grained detection of a specific emotion or emotion-related affective states featuring guilt.

Due to its popularity, there exist a considerable number of datasets on Emotion Detection available publicly and featured in numerous studies, such as Vent^17^, XED^18^, GoEmotions^19^, EmotionLines^20^, Corpus of Emotion Annotated Suicide notes in English (CEASE)^13^, The “International Survey on Emotion Antecedents and Reactions” (ISEAR)^21^, deISEAR^22^, to mention a few. It is worth noting that many existing emotion detection datasets use emotion labels based on Ekman’s^23,24^ or Plutchik’s^25^ theory, or a variation thereof, often including the neutral label. In our study, we specifically focused on three datasets^13,17,21^ that contain instances of guilt.

**Vent dataset**^17^, a large annotated dataset of texts (texts are from 33M posts), emotions, and social connections from the Vent social media platform. In Vent, each post is associated with emotion, self-annotated with emotion by the post’s author at the posting time. In^17^ no experiments on ED were reported, but in^14^ the authors ran ED experiments using Naive Bayes (NB), Random Forests (RF) and Logistic Regression (LR), Deep Neural Networks and Bi-LSTM. They employed two sets of feature representations, based on the type of method. For each of the traditional ML methods, they ran two sets of experiments, one using Bag-of-Words and then using TF-IDF feature vectors. The same procedure was used for Neural Network methods, but now using FastText and BERT embeddings as features. This resulted in ten (10) combinations of methods and features, with their best model achieving 19% and 21% in average macro and micro F1-score respectively, which is not surprising, considering the number of classes in Vent.

The *CEASE dataset*^13^, consisting of 2393 sentences extracted from around 205 English language suicide notes, collected from various websites and annotated for 15 emotion classes. For their experiments, they first employed three deep learning (DNN) models (Convolutional Neural Network^26^, Long Short Term Memory^27^, and Gated Recurrent Unit model^28^), the three DNN models were later combined to create two additional models (one majority vote ensemble and one multi-layer perceptron-based ensemble model). For comparison purposes, they trained and tested various combinations of traditional ML classifiers (Multinomial-NB, RF, LR, and Support Vector Machine (SVC)) with different sets of features. The MLP ensemble and LTSM models achieved the best results, with an F1-Score of 59% on average for all classes, and 48% for *guilt*, 4 points less than the performance of the majority vote ensemble on this particular class. Among the traditional ML models, LR was the best-performing model, with an average F1-score of 48% and 35% for the guilt class.

The *ISEAR dataset*^21^, a collection of 7669 sentences extracted from questionnaire answers from respondents from different countries, in different languages, as part of the ISEAR project in the ’90s, the sentences were first translated and then annotated for 7 emotion classes. This dataset inspired the creation of two new datasets, the enISEAR (English ISEAR) deISEAR and (Deutsch ISEAR)^22^, both comprising 1001 descriptions obtained from the study participants after being presented with emotion and asked to describe an event in which they felt that particular emotion. Both datasets have the same number of descriptions, but are different in length, thus resulting in deISEAR being divided into 1084 sentences and a vocabulary size of 2613 distinct tokens, and enISEAR into 1366 sentences and a vocabulary of 3066. In^22^ the authors trained a Maximmum Entropy (MaxEnt) classifier with unigram features on ISEAR^21^ and applied it to enISEAR and a translated version of deISEAR, achieving an average micro F1-Score of 47%, on both datasets, 42% on guilt class on deISEAR, and 41% for the guilt class on enISEAR.

## Dataset  development

For this research, three existing datasets were used as a starting point: the Vent^17^, ISEAR^21^ and CEASE^13^  Emotion datasets. The selection of these specific datasets was mainly for two reasons, (1) they are each from a different domain (source of where the texts were collected), and (2) they all contain guilt as one of the classes. A full description is provided below.

### Dataset preparation

Initially, we considered all samples from each dataset because the Vent dataset contains 33M samples, we only selected samples from the feelings category, which is the category that contains the guilt subclass. This first sampling resulted in 4,358,680 samples from Vent, 7666 from ISEAR, and 2393 from CEASE. Table 1 shows the emotion distribution based on this selection. Emotion classes that are present in at least two original data sources are labeled with the original name, and those only appearing in one data source are labeled as “others” in the table. As shown in Table 1, there were a total of  4,368,739 samples, of which 135,601 were texts labelled with guilt, and 4,233,138 with joy/happiness, anger, sadness/sorrow, fear, disgust, shame, and other emotions.

**Table 1:** Initial Data sampling result.

	Dataset		
Emotion	Vent	ISEAR	CEASE
Guilt	134,434	1093	74
Joy/happiness	–	1094	38
Anger	–	1096	79
Sadness/sorrow	–	1096	305
Fear	–	1095	29
Disgust	–	1096	–
Others	4,224,246	–	1868
Shame	–	1096	
Total	4,358,680	7666	2393

 With the initial selection, we proceeded with a second round of selection, to deal with the enormous differences in size. For this, we first limited the choice of non-guilt samples to the number of guilt samples found in each original dataset, giving us 135,604 guilt and 135,604 not-guilt samples, totaling 271,208 samples.

Because we are dealing with data from three different sources, we consider a few different steps for cleaning the data, taking into account its origin. The following steps were taken to identify and fix problems with the dataset:

The Vent dataset was obtained through an automated process of scraping millions of social media posts. Given the nature of this data collection method, it is reasonable to expect that there may be certain issues with the dataset. Upon initial inspection of the text samples, it became apparent that some of them were composed of only a single character space, special character, or emojis. After identifying these unhelpful samples, the next step in the cleaning process was to identify and remove duplicated text-label combinations, duplicated texts, and texts in languages other than English. For the ISEAR dataset, since it was compiled from questionnaire answers, we checked whether there are samples with no “no response”, “blank” or similar values, which are a clear indication of the absence of an answer from the respondents.

### VIC dataset statistics

With the dataset fairly cleaned, we proceeded to sample all guilt instances from each dataset and set the returned number of samples as a cap for selecting non-guilt samples, which resulted in 245,826 samples, which were then binarized (all non-guilt labels converted to “no guilt”), followed by a random selection of up to 1200 samples per class per origin, which led to the final dataset comprised of 4622 samples, with a balanced class distribution.

**Table 2:** A sample of texts and labels, with origin label from dataset VIC.

Text	Origin label	Binary label
Why do I always go back to christian why does he accept every time I fuck up and go back to him	Guilt	Guilt
Its two am and all I can think about is you I want you here I need you now I dont think I can ever walk away from you I wish I could move on but how do you move on from something youve never really had ugh	Tired	No guilt
My mom brought me and sister home because we were hungry and tired so I’m back in my nice bed	Sleepy	No guilt
Vent would be more useful if I could actually use words to describe my feelings	Tired	No guilt
on embarking on university life I came from a different city and did not know anybody at the uni I was frightened because my well known and loved friends also all my security had been taken away	Fear	No guilt
Fear paralysing that I would not be accepted by the god who I believed to be there because I was morally bankrupt before becoming a christian and realising that that was why christ came to free us from sin and to forgive us	Fear	No guilt
I am sorry to the people that I love but I can not fucking take it anymore	Guilt	Guilt
On a day like this everyone seeks the company of beloved ones	Love	No guilt
I am sorry once again	Guilt	Guilt
I angered a close friend and he was injured	Guilt	Guilt

**Figure 1:**
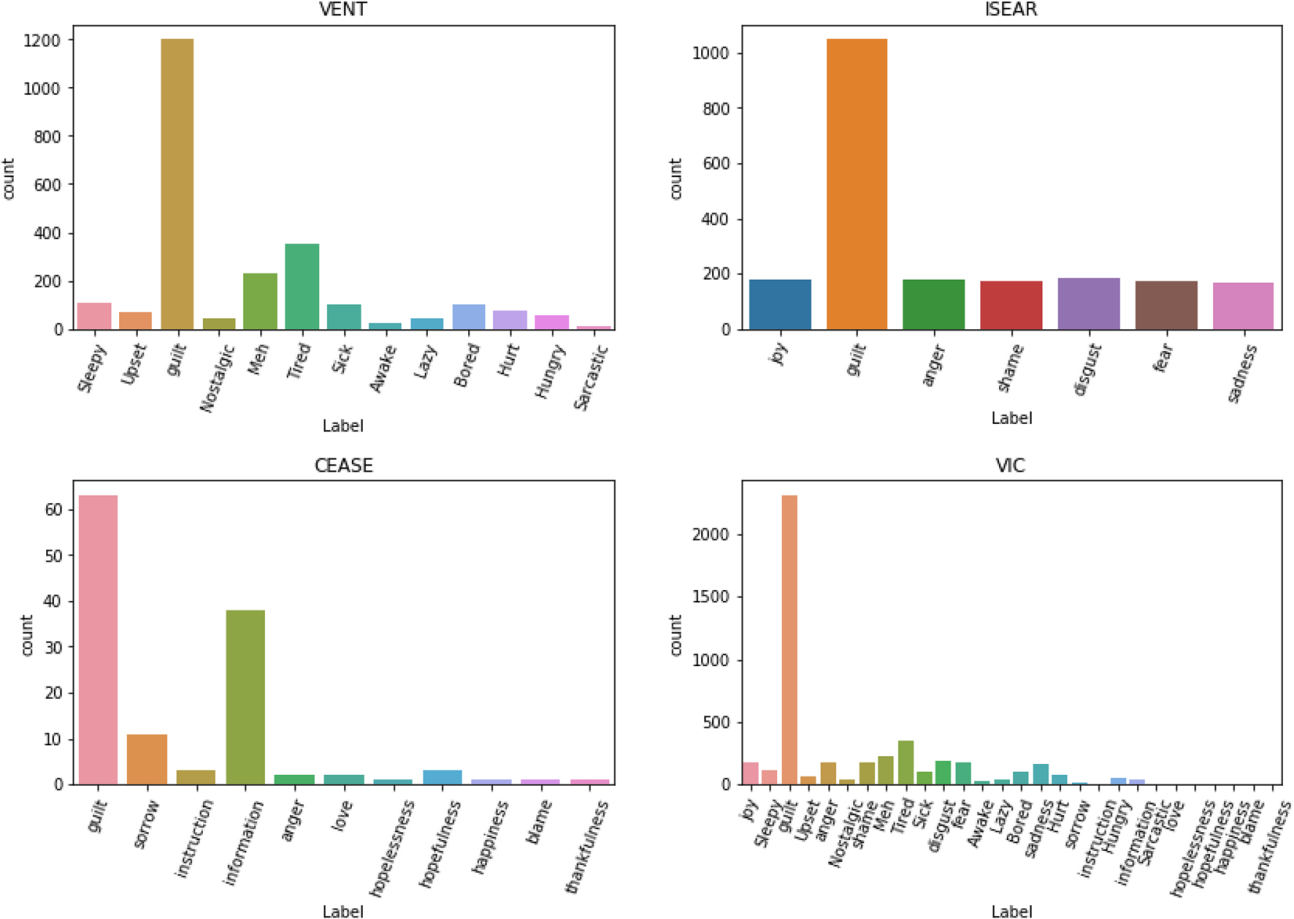
Original label distribution for each subset.

Our resulting dataset (VIC) contains 126 samples from CEASE, 2096 from ISEAR, and 1248 from Vent, Table 2 presents a sample of texts in VIC and Table 3 details the label distribution by sample origin and Fig. 1 depicts the distribution of non-guilt class samples distribution for each origin and their distribution in VIC. As shown in Table 3, initial data exploration revealed that guilt samples generally have a slightly smaller average word length when compared to the non-guilt samples, and the largest texts are of non-guilt samples.

**Table 3:** A summary of label and origin distribution in the resulting dataset VIC.

Class	# of Samples	Avg. Sent. Len.	Avg. Word Len.	Total words	Sample origin
Guilt	63	14.50	4.60	1914	CEASE
No guilt	63				
Guilt	1048	17.03	4.51	56054	ISEAR
No guilt	1048				
Guilt	1200	15.92	4.44	94179	Vent
No guilt	1048				

## Benchmark experiments

To provide a baseline for the guilt detection task, we conducted two sets of experiments, one using traditional machine learning and the second using neural network-based methods. For the traditional machine learning methods, we used the scikit-learn library^29^, and Keras library^30^ for the neural network-based methods. For the traditional machine learning methods, we first conducted hyperparameter tuning using a Grid Search algorithm and then trained and tested models with the best parameters from the grid search which was then compared with the results from neural networks models without tuning them. The full details of the experiments are detailed in the following subsection. To properly assess the effects of the dataset origin in the task, for each combination of method and features, we tested on subsets of the dataset based on the sample origin and finally on the whole dataset.

### Methods

*Traditional machine learning* we have chosen three methods, namely Support Vector Machine (SVM) a machine learning algorithm widely used in classification tasks. It has the property of being able to learn the correct labeling of data points that belong to different classes, given a set of training examples. Multinomial Naive Bayes, a Bayesian learning approach also widely used in natural language processing tasks, and Logistic Regression, a binary classifier that uses sigmoid activation in the output layer to predict the label. For each method, we experimented using two different features type, BoW and tf-idf vectors.

*Neural networks* for neural network methods, we selected Convolutional Neural Networks and inspired by the experiments in^31,32^, we added Bi-directional Long Short Term Memory (BiLSTM) to our choices of neural network models. All of our Sequential models start with an Embedding layer, with 64 dimensions and the vocabulary and input length based on the training data, with a final Dense layer with sigmoid activation.

- CNN is a feed-forward neural network called commonly used to analyze visual images by processing data in a grid-like architecture. According to^32^ CNNs are able to automatically learn features from the input text and have been successful in many natural language processing tasks. in addition to the layers in common to all the other models, our CNN has a 1-D Convolutional layer, a Global Max Pooling layer, and a Dense layer with relu activation.
- Bi-LSTM^33^, a form of recurrent neural network that uses LSTM cells (i.e. cell state representations) to adaptively change the size of its step length between input. This mechanism makes it possible for the network to learn long and short-term dependencies, as well as dynamics that span multiple time steps and hidden states. this NN is made of a Bidirectional LSTM layer and Dropout layers.

### Feature engineering

*Bag of Words* each instance of text in our corpus is treated as a collection of words, that is then vectorized using a Count Vectorizer using sklearn library’s feature extraction package, with n = {1-4}.

*TF-IDF* as a second feature option, we used the n-gram term frequency and inverse document frequency Vectors with n = {1-4},

### Hyperparameter tuning with GridSearch 

We used TF-IDF as features and trained classical ML classifiers for the first set of experiments. We used GridSearch to find the best hyperparameters (C for SVM and LR and alpha for MNB for example) and feature combinations for each ML model. The entire hyperparameter search space is presented in Table 4.

**Table 4:** Hyper parameter search configuration.

Method	Hyperparameter	Search space
LR	Dolver	[newton-cg, lbfgs,sag, liblinear, saga]
	C	[0.01, 0.1, 1, 10, 100]
	Penalty	[’l1’, ’l2’, ’elasticnet’]
SVC	Kernel	[linear, rbf]
	C	[0.01, 0.1, 1, 10, 100]
	Gamma	[0.001, 0.0001]
GB	n_estimators	[16, 32]
	Learning_rate	[0.8, 1.0]
DT & RF	max_depth	[5,10,25,None]
	min_samples_leaf	[5, 10, 20, 50, 100]
	Criterion	[gini, entropy]
RF	n_estimators	[16, 32]
MNB	Alpha	[0.1, 0.2, 0.3, 0.4, 0.5, 0.6, 0.7, 0.8, 0.9, 1.0]
	Fit_prior	[True, False]
	Class_prior	[None, [.1,.9],[.2, .8]]

### Dataset experiment setup

*Origin-based subsetting* We selected the samples from VIC based on their origin, resulting in three sub-datasets, and ran experiments on each of them separately, using the standard train and test split, with 10% samples for testing and 90% for training each time.

*Combined and Shuffled*with the help of the pandas library, we shuffled the entire VIC dataset and conducted experiments on it, also using the 90-10 train and test splits.

*Leave-one-origin-out training and testing* In addition to the experiments detailed above, we ran train and test experiments using examples from two origins for training and the third one for testing.

### Ethics statement

The research presented in this manuscript involves using three different datasets, namely the ISEAR, CEASE, and Vent datasets. All ethical considerations for each dataset were addressed in each paper that initially introduced them. The dataset comprises texts of three types, social media posts, suicide notes, and psychological study questionnaire answers. The resulting dataset used in this study only contains texts and labels, with no identifying or sensitive information. The authors obtained access to the datasets through reasonable requests to each of the dataset’s authors/creators and fulfilled and followed all the compliance requests set forth by the authors. The authors also ensured that identifying and sensitive information was removed from the resulting dataset to protect the individual’s privacy. The resulting dataset is available upon reasonable request due to compliance constraints on some of the samples because of their origin.

## Results and discussion

We started by running a hyperparameter search for the traditional ML models (which resulted in 478 candidate model configurations, from combining methods, hyperparameters, and features) on the VIC dataset. The resulting models were then ranked based on their overall performance, and for each method, we selected their best parameters for the subsequent tests. As shown in Table 5, for the tf-idf feature type, the best performing algorithm is MNB with an F1-score of 0.72, using alpha = 0.9, class_prior = None, and fit_prior = True. LR and SVC also have similar performances, with F1 scores of 0.68 and 0.67, respectively. The best-performing RF and DT have F1 scores of 0.67 and 0.65, respectively.

For the Bow feature type, MNB also has the highest F1-score of 0.72, using alpha = 0.7, class_prior = None, and fit_prior = True. LR has an F1-score of 0.69, while SVC has an F1-score of 0.67. The best-performing RF and DT have F1 scores of 0.67 and 0.66, respectively. The best-performing GB has an F1-score of 0.63, using n_estimators = 32 and lr = 1.

Table 5 shows the hyperparameter results for different machine learning algorithms and feature types. The evaluation metric used is F1-score, and the hyperparameters selected by the tuning process are listed as well.

**Table 5:** Hyperparameter search results for different machine learning algorithms and feature types using F1-score as the evaluation metric.

Features	Estimator	Min	Mean	Max	Std dev	Hyperparameters
Tf-idf	Multinomial Naive Bayes	0.71	0.72	0.75	0.015	$\alpha =1.0$, class_prior = None, fit_prior = True
	Support Vector Machine	0.68	0.69	0.71	0.009	$C=100$, kernel = linear
	Logistic Regression	0.68	0.69	0.70	0.007	$C=100$, penalty = l2, solver = saga
	Random Forest	0.33	0.58	0.67	0.127	$n\_estimators=32$, criterion = gini, max_depth = None, min_samples_leaf = 100
	Gradient Boosting	0.60	0.62	0.64	0.014	$n\_estimators=32$, lr = 0.8
	Decision Tree	0.60	0.61	0.63	0.010	criterion = entropy, max_depth = None, min_samples = 10
BoW	Multinomial Naive Bayes	0.70	0.71	0.73	0.011	$\alpha =0.7$, class_prior = {0.1,0.9}, fit_prior = True
	Logistic Regression	0.65	0.67	0.69	0.016	$C=10$, penalty = l2, solver = sag
	Random Forest	0.47	0.61	0.69	0.079	$n\_estimators=32$, criterion = gini, max_depth = None, min_samples_leaf = 10
	Support Vector Machine	0.65	0.66	0.67	0.008	$C=10$, kernel = linear
	Gradient Boosting	0.63	0.65	0.67	0.014	$n\_estimators=32$, lr = 0.8
	Decision Tree	0.52	0.57	0.62	0.034	criterion = entropy, max_depth = None, min_samples = 10

### Experiments on VIC dataset

Based on the Hyperparameter search results detailed in Table 5, we again trained and tested twelve (12) classical ML classifiers with their best-performing hyperparameters, using a K-Fold cross-validation strategy, with *k = 5*, thus training on 80% of the data and testing on the remainder 20%, on each fold. Multinomial Naive Bayes achieved the best performance, 72% F-Score when trained using tf-idf vectors as features and 71% with BoW feature vectors, as expected, as per the result of our hyperparameter tuning. Additionally, we ran tests using neural network classifiers, a CNN and BiLSTM as described in the previous section, equally trained and tested with the same K-Fold strategy as our classical ML models. Our CNN model achieved an F1-Score of 68%, 4% less than our best-performing MNB, which can be attributed to the fact that the proposed NN models were not subject to any fine-tuning. Regardless of the feature used, RF models had the worse performance amongst all the classifiers we experimented on, with an F1-Score of 52% overall. In general, tf-idf feature vectors gave better results for all methods when compared with BoW which is in line with the findings of previous research, with the only exception being the RF classifier and the detailed results are depicted in Table 6.

**Table 6:** Detailled results of different combinations of ML methods and features on the entire VIC dataset.

Feature	Model	Acc	Precision	Recall	F1-Score
Traditional machine learning models					
tf-idf	DT	0.61	0.62	0.61	0.61
	RF	0.52	0.53	0.75	0.58
	MNB	0.68	0.64	**0.83**	**0.72**
	LR	**0.70**	**0.72**	0.67	0.69
	GB	0.65	0.68	0.56	0.61
	SVC	**0.70**	**0.72**	0.67	0.69
BoW	DT	0.62	0.63	0.59	0.61
	RF	0.53	0.55	0.59	0.52
	MNB	0.66	0.62	**0.83**	**0.71**
	LR	0.66	0.66	0.69	0.67
	GB	0.65	**0.70**	0.52	0.59
	SVC	**0.68**	**0.70**	0.63	0.67

Neural network models					
Text tokens	CNN	**0.68**	**0.67**	**0.70**	**0.68**
	bilstm	0.64	0.64	0.66	0.64

Significant values are in bold.

### Origin-based subsetting

To better understand the effects of sample origin on the results, we conducted training and testing on each subset of our data based on the sample origin. For each method+feature combination, on each subset, we calculated the accuracy, precision, recall, and F1 scores, which are presented as summarized in Table 7. This result tells us that:

**Table 7:** Performance of different combinations of ML methods and features on every origin subset.

		Vent				ISEAR				CEASE			
Feature	Model	Acc.	Prec.	Rec.	F1-Score	Acc.	Prec.	Rec.	F1-Score	Acc.	Prec.	Rec.	F1-Score
Traditional machine learning models													
tf-idf	**DT**	0.59	0.59	0.58	0.59	0.62	0.63	0.61	0.62	0.68	0.73	0.62	0.66
	**RF**	0.52	0.54	0.46	0.45	0.52	0.22	0.26	0.24	0.49	0.40	0.80	0.53
	**MNB**	0.66	0.62	0.82	**0.71**	0.72	0.69	0.82	**0.75**	0.77	0.72	0.91	**0.80**
	**LR**	0.68	0.69	0.65	0.67	0.73	0.75	0.71	0.73	0.75	0.71	0.84	0.77
	**GB**	0.64	0.67	0.55	0.60	0.67	0.70	0.61	0.65	0.79	0.91	0.65	0.75
	**SVC**	0.68	0.69	0.64	0.67	0.73	0.74	0.71	0.73	0.76	0.84	0.65	0.73
BoW	**DT**	0.62	0.63	0.60	0.61	0.65	0.66	0.63	0.64	0.72	0.75	0.69	0.71
	**RF**	0.52	0.54	0.42	0.43	0.51	0.20	0.15	0.13	0.49	0.40	0.80	0.53
	**MNB**	0.64	0.61	0.82	**0.70**	0.70	0.66	0.83	**0.74**	0.60	0.56	0.95	0.71
	**LR**	0.65	0.64	0.71	0.67	0.73	0.73	0.72	0.73	0.75	0.74	0.75	**0.74**
	**GB**	0.66	0.72	0.52	0.61	0.69	0.73	0.61	0.66	0.76	0.90	0.60	0.71
	**SVC**	0.66	0.68	0.60	0.64	0.73	0.74	0.71	0.72	0.73	0.82	0.59	0.68

Neural network models													
Text tokens	**CNN**	0.63	0.68	0.61	**0.66**	0.71	0.71	0.73	**0.71**	0.74	0.74	0.77	**0.74**
	**BiLSTM**	0.64	0.64	0.65	0.65	0.65	0.65	0.67	0.67	0.66	0.69	0.68	0.67

Significant values are in bold.

- On the Vent subset, MNB with BoW feature vector outperformed all other models, with an F1-Score of 71%, followed by MNB with tf-idf and CNN models, with 70% and 66% F1-Score, respectively. Here RF was the worst-performing classifier, regardless of the features used.
- On ISEAR subset, both MNB+tf-idf and MBN+BoW achieved highest F1-Scores of 75% and 74% respectively, but MNB+BoW however achieving a better recall. Our CNN model achieved an F1-Score of 71%, and the RF classifier exhibited the worse results.
- For the CEASE subset, the MNB classifier trained with tf-idf features achieved an F1-Score of 80%, followed by GB with tf-idf and the CNN model with 75% and 74% F1-Score respectively.

From Table 7 we can conclude the following:

1. Overall, classifiers with the best hyperparameters tuned on VIC achieved better when trained and tested on the smaller subset (CEASE), and on average, they performed a little better on each subset than in the overall dataset, telling us that the models are fairly good at generalizing and that they are more suitable for short texts classifications. Note that the average sample length in Vent is higher than in ISEAR which is higher than in CEASE.
2. Every model performed better with tf-idf, compared to BoW feature vectors across all subsets, except for RF ( with virtually the same result regardless of the feature type) and LR and GB where in general, training with BoW feature vectors.
3. At first glance, similarly to the classical ML classifiers, for CNN models the shorter the sample texts length the better the performance, while BiLSTM seems unaffected by the sample text length.

### Leave-one-origin-out training and testing

When leaving one subset for testing and using the other two for training, as depicted in Table 8, the results show that models trained on VENT+ISEAR perform better, in almost all instances of train and testing, except the SVM model with Bag-of-Words, which showed a poor F1-score (45%). These results can naively be explained by the fact that VENT+ISEAR models benefited from having a large number of training samples (~ 4.5 K) and greater average sample length than those in the testing set (CEASE subset).

**Table 8:** Summarization of the best performing classical ML models using different combinations of two subsets for training and one for testing.

Feature	Model	Test Set											
		Vent				ISEAR				CEASE			
		Acc	Precision	Recall	F1-Score	Acc	Precision	Recall	F1-Score	Acc	Precision	Recall	F1-Score
tf-idf	**DT**	0.56	0.57	0.45	0.50	0.57	0.60	0.42	0.50	0.65	0.63	0.71	0.67
	**RF**	0.50	0.50	1.00	**0.67**	0.50	0.50	1.00	**0.67**	0.48	0.36	0.06	0.11
	**MNB**	0.57	0.55	0.82	0.66	0.57	0.54	0.89	**0.67**	0.61	0.57	0.90	0.70
	**LR**	0.58	0.56	0.66	0.61	0.61	0.60	0.67	0.63	0.71	0.65	0.87	**0.75**
	**GB**	0.57	0.58	0.49	0.53	0.57	0.59	0.48	0.53	0.63	0.63	0.60	0.62
	**SVC**	0.57	0.56	0.63	0.59	0.61	0.60	0.65	0.62	0.68	0.64	0.86	0.73
BoW	**DT**	0.56	0.56	0.50	0.53	0.57	0.58	0.48	0.53	0.6	0.59	0.62	0.60
	**RF**	0.50	0.50	1.00	**0.67**	0.50	0.50	1.00	**0.67**	0.51	0.57	0.06	0.11
	**MNB**	0.53	0.52	0.89	0.65	0.54	0.52	0.93	**0.67**	0.52	0.51	0.89	0.65
	**LR**	0.55	0.56	0.52	0.54	0.58	0.60	0.51	0.55	0.67	0.63	0.79	**0.70**
	**GB**	0.57	0.64	0.34	0.44	0.59	0.67	0.34	0.45	0.7	0.75	0.60	0.67
	**SVC**	0.58	0.61	0.43	0.51	0.57	0.61	0.40	0.49	0.67	0.67	0.70	0.68

Significant values are in bold.

## Limitations

Firstly, our dataset is relatively small, consisting of only 4622 samples. While it has a balance between the number of guilt and non-guilt samples, it however, does not have between the samples from different sources, a larger dataset would allow for more robust model training and testing. This could limit the generalizability of the models trained on this dataset, as they may not perform as well on larger datasets or different datasets with different characteristics. Secondly, the VIC dataset is primarily composed of samples from three sources (CEASE, ISEAR, and Vent). While this provides some diversity, it may not be representative of all possible sources of text related to guilt and non-guilt. This could as well limit the generalizability of the models trained on this dataset. To address this, one possible path for future work would be to consider expanding the dataset by collecting more samples or using transfer learning techniques to leverage larger datasets.

As for the baseline models in the present work, they only have two feature representations (tf-idf and BoW) and embeddings for the two neural network models (CNN and BiLSTM) to represent the text data. While these are commonly used techniques, there may be other feature representations or neural network models that could better capture the unique characteristics of the text data in the VIC dataset. In future work, we intend on experimenting with other feature representations and neural network architectures, including transformer-based models.

## Misclassification analysis

Error analysis is a crucial step in any NLP study, as it provides insight into the strengths and weaknesses of the model and helps identify areas for improvement. In this section, we present an analysis of some of the misclassified samples by our guilt detection models, in which we analyze the most common types of errors made by our models and attempt to understand the causes of these errors. By examining these errors, we hope to gain a deeper understanding of the nature of the task of detecting guilt in text and to identify potential avenues for improving the performance of our models in future work. A sample of misclassified examples is presented in Table 9, and each instance of misclassification is explored in detail in this section.

**Table 9:** A sample of misclassified examples from dataset VIC, using MNB+tf-idf.

Text	Label	Predicted
I really can’t anymore	No guilt	Guilt
Me: hey this person hasn’t talked to me in a bit my brain: they’re dead me: well no they could be driving or with friends or... my brain: they hate you and they’re dead me: no -my brain: THEYRE DEAD THEYRE DEAD THEYRE DEAD	No guilt	Guilt
My mother had for some time been trying to separate me from a ágood friend who, she thought, was not good company for me. áFinally, at breakfast one day, we had an argument and I tried to ádefend my friend.	No guilt	Guilt
A twitter Stan asked me if I have some pills to gargle Tried clicking on the notifications and it said they got deleted;)	No guilt	Guilt
Disordered eating implied This is harder than i anticipated considering how hungry we were ashfkdjsj camera-emoji	Guilt	No guilt
Someone told a lie that I had stolen his money.	No guilt	Guilt
Heard that my girl-friend was chosen for the English lectures and áI was not. I lost my temper and she is very upset now.	Guilt	No guilt
NSFW So I should have prepped my ass a lot more than that lmao	Guilt	No guilt
When two drug addicts tried to take away my money.	No guilt	Guilt
When one of my lovers told me that I was a flirt.	Guilt	No guilt

1. The text “I really can’t anymore” seems to express a feeling of exhaustion or despair, which could be interpreted as a negative emotional state and lead to a prediction of guilt. However, without more context, it is difficult to say for certain whether guilt is an appropriate label. It is possible that the model may have been influenced by the word “can’t” which could be interpreted as a form of guilt for not being able to handle a situation as could also be possible that the word “anymore” triggered the model to predict guilt, as it suggests some kind of frustration or negative emotion. It is also possible that the model may not have encountered enough examples of this type of language and therefore is not able to accurately predict the label.
2. “me: hey this person hasn’t talked to me in a bit my brain: they’re dead me: well no they could be driving or with friends or... my brain: they hate you and they’re dead me: no -my brain: THEYRE DEAD THEYRE DEAD THEYRE DEAD”. The predicted label is guilt while the actual label is no guilt
  - The model might have misinterpreted the use of all caps as an expression of strong negative emotions, leading to the prediction of guilt. However, in this context, the person is simply describing an internal dialogue, and there is no actual event that would cause guilt.
  - The sample seems to be a humorous or exaggerated representation of intrusive or anxious thoughts, so the predicted label of guilt may indicate that the speaker feels guilty for having these thoughts or for being anxious about their relationship with the person who has not contacted them.
3. “My mother had for some time been trying to separate me from a good friend who, she thought, was not good company for me. Finally, at breakfast one day, we had an argument, and I tried to defend my friend.” - The predicted label is guilt, while the actual label is no guilt.
  - The text describes a disagreement between the speaker and their mother over whether their friend is a good influence, but there is no indication of the speaker feeling guilty.
  - It is possible that the model picked up on the word “argument” and predicted guilt based on the assumption that arguments are usually associated with negative emotions. However, in this context, the person is simply defending their friend, and there is no indication of guilt.
  - This sample describes a conflict between the speaker and their mother over a friend. The predicted label of guilt may indicate that the speaker feels guilty for not being able to resolve the conflict or for causing their mother stress.
4. “A twitter Stan asked me if I have some pills to gargle...Tried clicking on the notifications and it said they got deleted;)”: The true label is “no guilt” and the predicted label is “guilt”. This sample is difficult to interpret without more context, but it may indicate that the speaker is being asked for a favor or being made an uncomfortable request. The predicted label of guilt may indicate that the speaker feels guilty for not being able to fulfill the request, but it is unclear why the model predicted guilt in this instance, as there is no indication of negative emotions or events.
5. “Disordered eating implied This is harder than i anticipated considering how hungry we were ashfkdjsj camera-emoji”: The true label is “no guilt” and the predicted label is “no guilt”.
  - The text expresses frustration about hunger, but there is no indication of guilt.
  - This sample seems to indicate that the speaker is struggling with disordered eating and finding it difficult to manage their hunger. The predicted label of no guilt may indicate that the speaker does not feel guilty about their struggle with disordered eating or their difficulty managing their hunger.
  - It is possible that the model did not pick up on the implication of disordered eating in this text, leading to misclassification.
6. “Someone told a lie that I had stolen his money.” - The predicted label is guilt while the actual label is no guilt. It is possible that the model picked up on the word “stolen” and predicted guilt based on the assumption that stealing is associated with negative emotions. However, in this context, the person is simply reporting an accusation.
7. “Heard that my girl-friend was chosen for the English lectures and I was not. I lost my temper and she is very upset now.”: The predicted label is “no guilt”, but the true label is “guilt”.
  - It’s possible that the model did not pick up on the guilt in the text, which is implied through the author’s loss of temper and the upset caused to the girlfriend.
  - The model may have also been confused by the use of the word “good” in the text, which could have made it think that the text was more related to “no guilt” than “guilt.”
  - The model may have failed to pick up on the implication that the person’s loss of temper was a negative event, leading to misclassification.
8. “NSFWSo I should have prepped my ass a lot more than that lmao” - The predicted label is no guilt while the actual label is guilt.
  - Here, the model could not pick up on the implication of a negative or embarrassing event, leading to misclassification.
  - A second possibility is that the model did not pick up on the guilt in the text, which is implied through the author’s use of a phrase that suggests a lack of preparation.
  - The model may have also been confused by the use of the NSFW tag (usually used to implicate something of mature or sexually explicit nature), which could have made it think that the text was more related to “no guilt” than “guilt”.
9. “When two drug addicts tried to take away my money.” - The predicted label is guilt while the actual label is no guilt.
  - The model may have incorrectly predicted guilt for this text due to the mention of “drug addicts” and “taking away my money.” It’s possible that the model assumed the author must have done something wrong to have encountered this situation when they were simply the victim of an attempted robbery.
  - Ambiguity: The text is brief and lacks a lot of context, so it’s possible that the author did something that could be interpreted as guilt-inducing in this situation. Perhaps they were carrying a large amount of money and were in an area known for drug activity, or maybe they responded to the situation with aggression or violence. And the lack of further context may have led the model to fail.
10. “When one of my lovers told me that I was a flirt” - The predicted label is ni guilt while the actual label is guilt. Possible reasons for the misclassification could be:
  - Ambiguity in the text: The phrase “one of my lovers” could indicate that the person has multiple partners, which might be interpreted as flirting. The classifier might have assigned a guilt label based on this ambiguity, while in reality, the person might not have been flirting at all.
  - Lack of context: The classifier might not have had enough information about the situation to judge accurately. The text doesn’t provide details about the nature of the relationship or the context of the accusation, which could have influenced the label.
  - Personal bias: The classifier might be influenced by personal bias or assumptions about what constitutes “flirting”. Due to the training data, the model might have learned a biased interpretation of the term “flirt” that doesn’t align with the writer’s intentions.
  In general, misclassification can occur for various reasons, including ambiguity in the text, errors in the training data or model, and differences in how people interpret or understand language. It’s important to continually evaluate and improve models to minimize misclassifications and improve accuracy.

## Conclusion

In this study, we developed a new binary classification dataset for guilt detection by extracting guilt and non-guilt samples from three existing multi-class emotion detection datasets. By doing so, we provided a unique resource for researchers and practitioners to study and develop machine learning models for guilt detection, a critical application in the fields of studies at the intersection of psychology and computational linguistics. We trained classical machine learning models and two neural network models, CNN and BiLSTM, to establish baselines using both tf-idf and BoW feature vectors.

Our results showed that, in general, every model performed better with tf-idf compared to BoW feature vectors, except for RF. LR and GB achieved better results when trained with BoW feature vectors. Moreover, CNN models performed better with shorter sample text lengths, while BiLSTM models were not affected by the text length.

Regarding subset-specific performance, on the Vent subset, the MNB model with BoW features achieved the highest F1-Score of 71%, outperforming all other models. On the ISEAR subset, both MNB with tf-idf and MNB with BoW achieved the highest F1-Scores of 75% and 74%, respectively, with the latter achieving a better recall. On the CEASE subset, the MNB classifier trained with tf-idf features achieved the highest F1-Score of 80%, followed by GB and the CNN model, achieving F1-Scores of 75

Overall, our study provides a valuable benchmark for future research in guilt detection. Our error analysis also revealed some of the weaknesses of our models, which could be addressed in future studies to improve performance. Our work highlights the importance of carefully selecting the training data and the choice of machine learning models when developing systems for guilt detection. Furthermore, our leave-one-out analysis demonstrated that our models can generalize to new data with reasonable accuracy.

## Data Availability

The ISEAR dataset is publicly available at https://www.unige.ch/cisa/index.php/download_file/view/395/296/ and the source paper introducing this dataset is cited correctly in this manuscript. The CEASE dataset is available upon reasonable request to the authors of the paper that first presented it, and a form for requesting access is available at https://www.iitp.ac.in/ ai-nlp-ml/resources.html. Portions of the Vent Dataset are publicly available, while the texts are only available upon reasonable request to the paper’s corresponding author who first introduced it. The resulting dataset used in this study, which only contains readers and labels, is also available upon reasonable request due to compliance constraints on some of the samples because of their origin.
